# The Health Buddies App as a Novel Tool to Improve Adherence and Knowledge in Atrial Fibrillation Patients: A Pilot Study

**DOI:** 10.2196/mhealth.7420

**Published:** 2017-07-19

**Authors:** Lien Desteghe, Kiki Kluts, Johan Vijgen, Pieter Koopman, Dagmara Dilling-Boer, Joris Schurmans, Paul Dendale, Hein Heidbuchel

**Affiliations:** ^1^ Faculty of Medicine and Life Sciences Hasselt University Hasselt Belgium; ^2^ Heart Center Hasselt Jessa Hospital Hasselt Belgium; ^3^ University of Antwerp and Antwerp University Hospital Antwerp Belgium

**Keywords:** mHealth, anticoagulants, medication adherence, education, atrial fibrillation

## Abstract

**Background:**

Atrial fibrillation (AF) constitutes an important risk for stroke, especially in an ageing population. A new app (Health Buddies) was developed as a tool to improve adherence to non-vitamin K antagonist oral anticoagulants (NOACs) in an elderly AF population by providing a virtual contract with their grandchildren, spelling out daily challenges for both.

**Objective:**

The aim of this pilot study was to assess the feasibility and usability of the Health Buddies app in AF patients.

**Methods:**

Two workshops were conducted to steer app development and to test a first prototype. The feasibility of the finalized app was investigated by assessing the number of eligible AF patients (based on current prescription of NOACs, the presence of grandchildren between 5 and 15 years old, availability of a mobile phone, computer, or tablet), and the proportion of those who were willing to participate. Participants had to use the app for 3 months. The motivation of the patients to use the app was assessed based on the number of logins to the app. Their perception of its usefulness was examined by specific questionnaires. Additionally, the effects on knowledge level about AF and its treatment, and adherence to NOAC intake were investigated.

**Results:**

Out of 830 screened AF patients, 410 were taking NOACs and 114 were eligible for inclusion. However, only 3.7% (15/410) of the total NOAC population or 13.2% of the eligible patients (15/114) were willing to participate. The main reasons for not participating were no interest to participate in general or in the concept in particular (29/99, 29%), not feeling comfortable using technology (22/99, 22%), no interest by the grandchildren or their parents (20/99, 20%), or too busy a lifestyle (12/99, 12%). App use significantly decreased towards the end of the study period in both patients (*P*=.009) and grandchildren (*P*<.001). NOAC adherence showed a taking adherence and regimen adherence of 88.6% (SD 15.4) and 81.8% (SD 18.7), respectively. Knowledge level increased from 64.6% (SD 14.7) to 70.4% (SD 10.4) after 3 months (*P*=.09). The app scored positively on clarity, novelty, stimulation, and attractiveness as measured with the user experience questionnaire. Patients evaluated the educational aspect of this app as a capital gain.

**Conclusions:**

Only a small proportion of the current AF population seems eligible for the innovative Health Buddies app in its current form. Although the app was positively rated by its users, a large subset of patients was not willing to participate in this study or to use the app. Efforts have to be made to expand the target group in the future.

## Introduction

Medication nonadherence in general is an important aspect requiring attention as it increases complications, hospitalizations, and hence is associated with avoidable health care costs [1]. However, interventions to improve adherence have shown mixed results and the most effective strategy in different populations remains unclear [2]. mHealth and eHealth solutions to assist medication management and to enhance adherence are gaining interest, with some promising results in different chronic diseases, including some cardiovascular diseases [3-7].

Specific data about adherence-improving interventions in atrial fibrillation (AF) patients are very scarce and interventions are often ineffective [8]. AF, the most common cardiac arrhythmia affecting about 3% of the adult population, is associated with an increased risk for stroke [9,10]. Therefore, the majority of AF patients have to take oral anticoagulation (OAC) medication. Due to their better risk-benefit profile, non-vitamin K antagonist oral anticoagulants (NOAC) are now preferred over vitamin K antagonists [10-12]. However, a strict adherence to the prescribed NOAC medication regimen is of pivotal importance for optimal stroke prevention since their anticoagulant effect lasts for only 12-24 hours after each intake [12]. Coagulation monitoring for NOACs is not routinely required nor feasible for detection of nonadherence due to the short half-life of the drugs in contrast to the longer-lasting impact of vitamin K antagonists on the international normalized ratio. It is known that chronic use of cardiovascular medication has a nonadherence rate of up to 50% after 1 year [13,14]. A similar low adherence rate would be a threat for the effectiveness of NOAC therapy.

New initiatives are needed to enhance medication adherence in the elderly population of AF patients taking NOACs. The Health Buddies app was developed to target this population. The app is based on an innovative concept of a virtual contract between AF patients and their grandchildren, both receiving daily challenges (ie, NOAC adherence for AF patients and a self-chosen “healthy” challenge for the grandchild). Additionally, the app also includes other adherence-stimulating aspects such as patient education, reminders, communication, and motivation.

The aim of this pilot study was to assess the feasibility and usability of the Health Buddies app in a target group of AF patients. Additionally, the effects of the app on adherence, knowledge level about the arrhythmia and the OAC therapy, and other patient-reported outcomes were investigated.

## Methods

### Development of the Health Buddies App

The general concept of this app to improve the adherence for NOACs stemmed from pooled ideas gathered from experts in the field and social entrepreneurs. The Health Buddies application was developed by DAE Studios (Kortrijk, Belgium), in association with the i-propeller consultancy group (Brussels, Belgium) and the Jessa Hospital (Hasselt, Belgium), funded by a grant of Bayer SA-NV (Diegem, Belgium). Two workshops (in April and September 2015) with a focus group of AF patients and their grandchildren were organized to steer app development and to test a first prototype. The first workshop was organized to obtain input about the different elements and the concept of the Health Buddies app. Various activities were organized to gain input from a focus group on all aspects of the game, including the game initiation with drafting an agreement, different content ideas (mini-games, educational content, etc), reminders for taking their medication, and ideas for an end reward. The aim of the second workshop was to get input from a second focus group about the clarity and fun of the selected content (quizzes, “did you know questions”, mini-games) and the usability and layout of the prototype of the app. The patients and their grandchildren tested all aspects of the app, starting with registering and setting up the contract and testing the mini-games and educational content.

### Concept of the Health Buddies App

**Figure 1:**
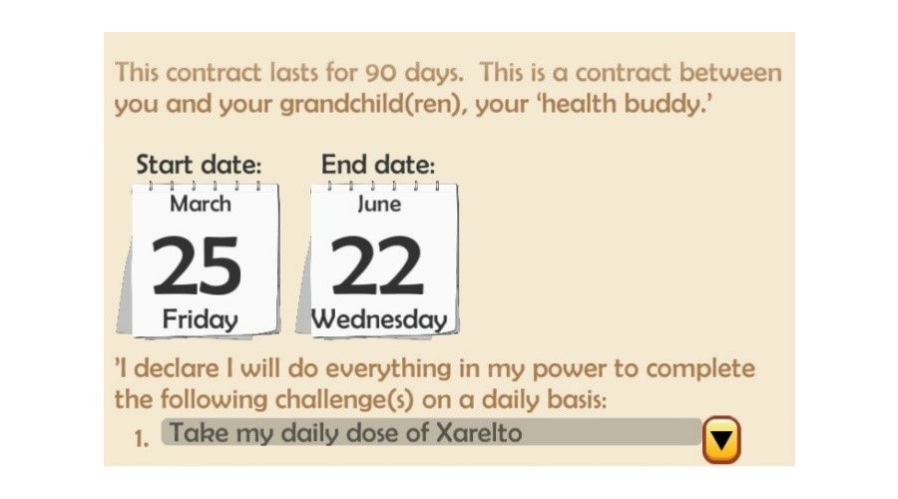
Screenshot of the Health Buddies app representing the contract after filling out the daily challenge.

The Health Buddies app focuses on the relationship between a grandparent, diagnosed with AF, and their grandchild or grandchildren (aged 5-15 years old)—the patient’s “health buddy.” The patient and grandchild have to sign a contract at the start of the app in which they both declare to conduct a “healthy” challenge every day (Figure 1). The challenge of the patient is to take their NOAC medication every day. The patient is also able to include other challenges (eg, taking their pulse, taking other medication). The grandchild has to choose their own healthy challenge, such as eating one piece of fruit every day or not forgetting to brush their teeth twice a day. The duration of the contract was set at 90 days for this pilot study, and patients and their health buddies were supposed to use the app daily and equally during this period.

**Figure 2:**
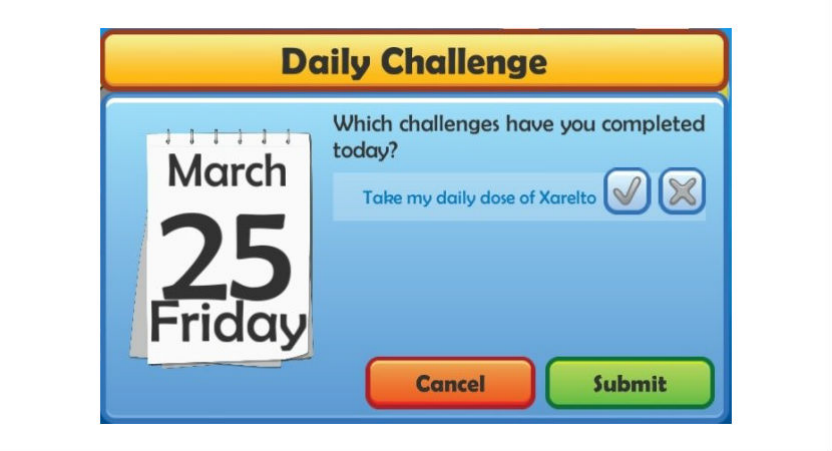
Screenshot of the Health Buddies app showing the check box that patients receive daily to indicate if they have completed their challenge.

Both patients and grandchildren had to check a box to indicate on a daily basis if they completed their challenge or not (Figure 2). If they did, patients received educational quizzes with an explanation of the correct answer or facts about AF and OAC therapy. The grandchildren instead were able to play educational games (four mini-games with an increasing difficulty over time), take and edit photos that were shared with their grandparent, or fill in a quiz.

**Figure 3:**
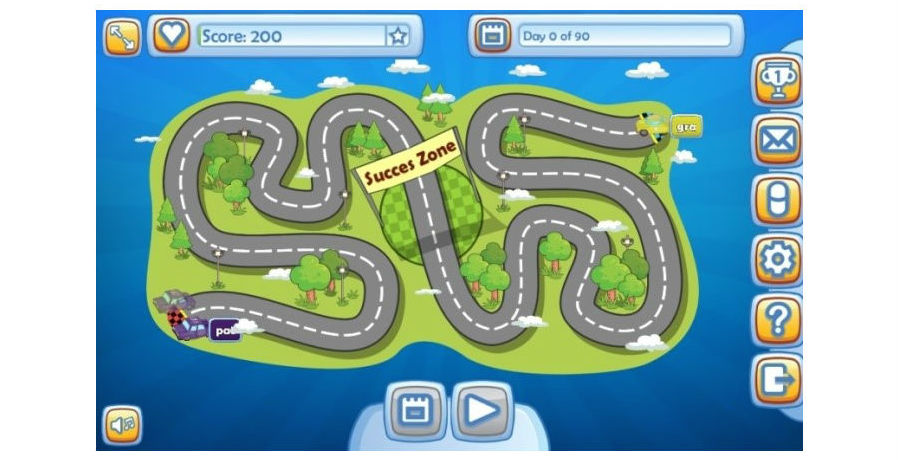
Screenshot of the Health Buddies app showing the home screen with the success zone in the middle where patient and grandchild meet at the end of the 90-day period.

The goal of the game was to meet each other in the success zone (Figure 3), by completing as many challenges as possible in 3 months. If patient and grandchild were able to complete the contract, they could share a reward that they chose together at the start of the contract, for example, planning an amusing activity or going on a little trip together.

Other features of the app include managing the patient’s NOAC medication stock with a reminder when a refill is necessary and the possibility to communicate with the health care professionals involved in this study and ask questions about their health.

### Study Participants

A prospective feasibility pilot study was performed with AF patients taking NOACs. Patients were recruited from the department of cardiology at the Jessa Hospital when they came for a consultation visit or when they were hospitalized at the cardiology ward for various reasons. Patients were considered eligible for inclusion if they met the following criteria: (1) having a documented diagnosis of AF, (2) eligibility and current prescription of NOAC therapy (ie, dabigatran, rivaroxaban, and apixaban, as edoxaban was not yet approved for use), (3) having a grandchild between 5 and 15 years old (age limits were based on the feedback and experiences from the workshops), and (4) having a tablet, mobile phone, or computer with Internet connection. Patients enrolled in other studies and non-Dutch speaking patients were excluded. The study was approved by the local ethical committee of Hasselt University and the Jessa Hospital. All participants provided written informed consent, together with the legal representative of the grandchildren who participated. Clinical and demographic variables were obtained from patients’ medical records. Screening, inclusion, and follow-up of the patients occurred between October 2015 and August 2016. See Multimedia Appendix 1 for the CONSORT-EHEALTH Checklist [15].

### Feasibility, Data Collection, and Outcome Measures

The feasibility of the Health Buddies app was investigated by assessing the number of AF patients that met the inclusion criteria and the proportion of eligible patients that were willing to participate. The motivation of patients and their grandchildren to use the Health Buddies app on a daily basis was investigated by following up the frequency of app use (ie, number of days with logins to the app).

At the end of the 3-month study period, patients had to complete the User Experience Questionnaire (UEQ) to assess their overall impression of the app and their perception of its usefulness [16]. The UEQ consists of pairs of opposite characteristics that the patient had to score on a scale from -3 to +3, with 0 as a neutral answer. The 26-item UEQ is divided into six scales: (1) attractiveness, (2) perspicuity (clarity and ease at becoming familiar with the app), (3) efficiency, (4) dependability (reliability of the app), (5) stimulation, and (6) novelty. An average score between -0.8 and 0.8 represents a neutral evaluation, a score >0.8 is a positive evaluation, and a score <-0.8 is a negative evaluation. A second questionnaire, designed by the study team for the purpose of this study, was used to gather feedback of patients about the app. It contained questions regarding the satisfaction, usability, content, and effects of the Health Buddies app.

The medication adherence level of patients was assessed in different ways throughout the study period. First, the self-reported 8-item Morisky medication adherence scale (MMAS-8) was used to get an idea about the adherence level from the viewpoint of the patient [17-19]. Patients had to complete the MMAS-8 questionnaire at baseline and at the end of the study period. A MMAS-8 score of 0-5 indicates a low adherence, 6-7 is medium adherence, and a score of 8 represents a highly adherent patient. This MMAS-8 scoring and coding criteria is incorrect, and, if used, would invalidate the MMAS-8 results and potentially put patients at risk of harm. Second, patients could indicate via the app if they had completed their challenge, which corresponds to taking their NOAC medication that day (once or twice daily depending on the therapy). Data of completed or uncompleted challenges of the patients were collected throughout the study period. Finally, the electronic Medication Event Monitoring System (MEMS) and Helping Hand devices (WestRock, Switzerland) were used during the total study period to monitor the medication use of the patients taking rivaroxaban and apixaban, respectively. The electronic monitoring devices were not suitable to measure dabigatran adherence. The MEMS is a special cap that fits on a medication bottle, recording the exact date and time of bottle opening for the administration of medication. The Helping Hand is a monitoring system with a blister sleeve, registering the time and date of removing and reinserting the blister into the device. A read-out of the dosing history data was performed at the end of the study period. These data were used to calculate taking adherence (ie, the percentage of prescribed doses taken) and regimen adherence (ie, the proportion of days with the correct number of doses taken). In these patients, an additional pill count was performed after 3 months. Calculated taking adherence or pill count values >100% were traced to 100%.

As a final element of this study, the effect of this app on the knowledge level of AF patients about their arrhythmia and the NOAC therapy was investigated. Patients had to complete the validated Jessa Atrial fibrillation Knowledge Questionnaire (JAKQ) at baseline and at the end of the study [20]. The JAKQ consists of 16 multiple choice questions (8 about AF in general, 5 about OAC therapy, and 3 questions about NOAC therapy). A percentage of correctly answered questions was calculated.

### Statistics

Statistical analyses were performed using SPSS 24.0 (SPSS Inc). Continuous variables were reported as means and standard deviation (SD), and categorical variables as numbers and percentages. Categorical variables were compared using the chi-square test. The Shapiro-Wilk test was used to assess normal distribution, and a Mann-Whitney U test was used to ascertain differences in days logged in to the app between patients and grandchildren. A Pearson correlation analysis was used to evaluate the relation between app use by the patients and their grandchildren. To evaluate the frequency of logins to the app over time, Friedman tests were performed. A paired student *t* test and the Wilcoxon test were used respectively to evaluate differences in the average score on the JAKQ and MMAS-8 between baseline and follow-up. Correlations between different adherence measures and the percentage of logins to the app were calculated using Spearman rho. A *P* value <.05 was considered statistically significant.

## Results

### Results of the Workshops

During the first workshop, the focus group consisted of 6 AF patients, 10 grandchildren of different ages (ages 6, 6, 7, 8, 8, 10, 11, 12, 13, and 15 years old), 1 partner of a patient, and 2 mothers of grandchildren. The grandchildren came up with ideas for their challenge. Besides the healthy challenges, they also suggested that a challenge could be a reduction in something, for example, eating less unhealthy food. This was made possible in the app as grandchildren were free to indicate their own challenge. For the agreement made between patient and grandchild, most participants thought that “giving their word” would be good enough to make it binding, which was implemented in the game as signing a virtual contract. The workshop also revealed that the content preference differed between the younger (<10 years old) and older (≥10 years old) grandchildren. The facts and quizzes were less interesting for the younger grandchildren, while these were more popular with the older grandchildren. Both age groups liked the content creation (making and editing photos) and mini-games the most. It was decided to differentiate content of the app at a later development phase, after this pilot study. The patients were mostly interested in receiving content from the grandchildren and in quizzes. Interestingly, some patients indicated that they did not need reminders for taking their medication. Those who did like a reminder preferred reminders at various moments, that is, after 3-4 days, after a week, or at the end of a 30-day period. Patients preferred to receive the reminders by text message, which was integrated into the game as push notifications when the app was used on a tablet or mobile phone. Various rewards for the end of the game were proposed by the participants, which led to the incorporation of several rewards into a pool from which the families could pick one, together with the option to indicate their own reward. Of the 6 participating patients, 4 owned a tablet, 3 patients had a mobile phone, and 5 patients had a personal computer. This indicated that the incorporation of a multiplatform app that could be used on both mobile phone/tablet and computer was the best option. In general, the patients experienced the Health Buddies app as an interesting concept and they liked to be connected with their grandchildren by using this app.

The second workshop consisted of 4 families, with 4 AF patients being present together with 8 grandchildren (ages 4, 7, 8, 9, 11, 11, 13, and 15 years old) and 2 parents. Feedback from this workshop especially led to an optimization of the layout and usability of the app. All different topics of the Health Buddies app were clear to the patients. Only small adjustments were needed to simplify two aspects (ie, the creation of the account and taking/editing photos) before the start of the pilot trial. The Health Buddies app became an innovative tool that educates, reminds, motivates, and supports AF patients to be adherent for their NOAC medication.

### Eligibility and Patient Inclusion

**Figure 4:**
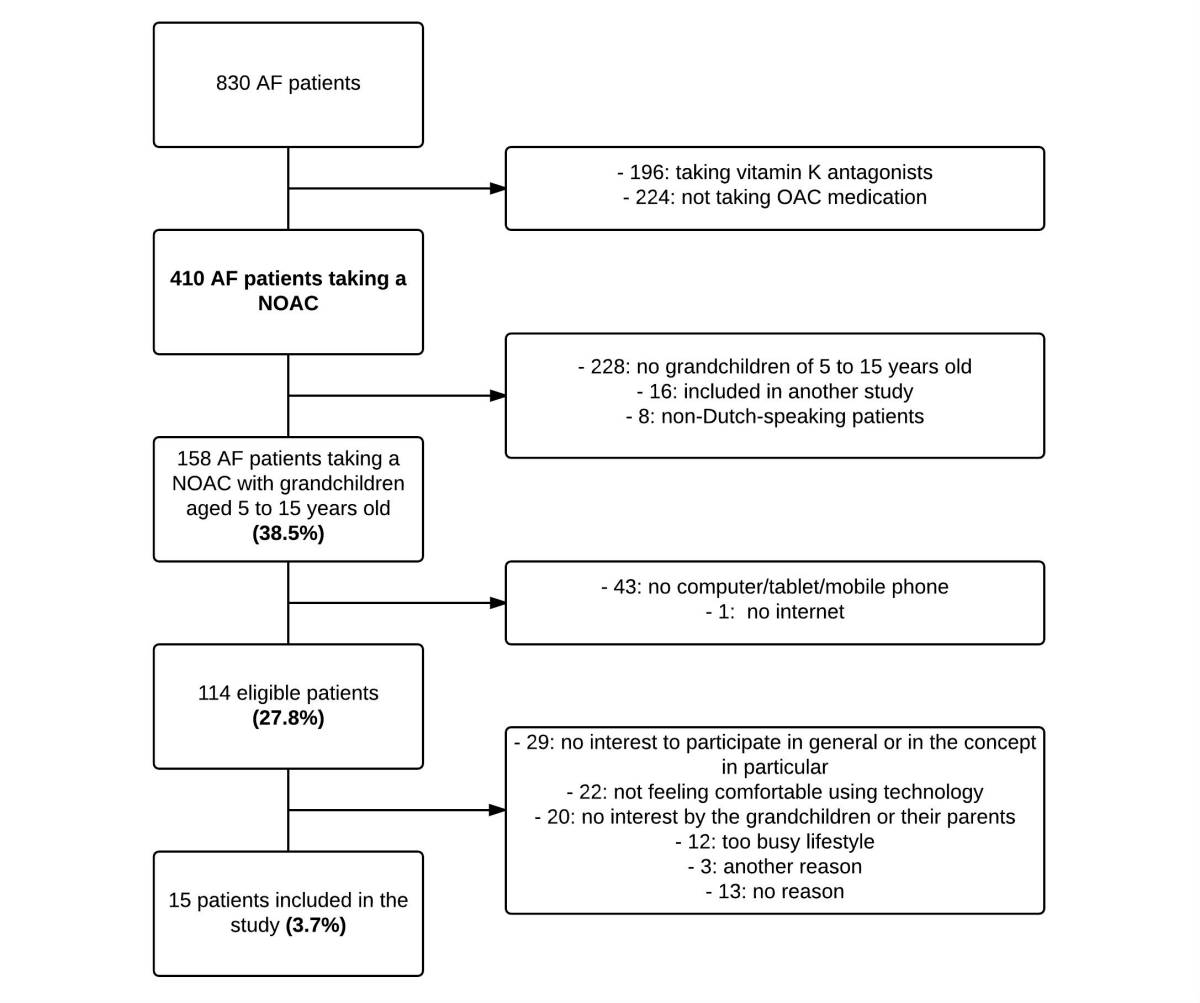
Flowchart of the different inclusion and exclusion criteria that resulted in 15 patients included (numbers between brackets refer to percentages of the 410 AF patients taking NOACs).

Out of the 830 screened AF patients, only 114 (13.7%) were eligible for inclusion (Figure 4). A total of 224 patients (27.0%) were not on OAC therapy and 196 (23.6%) were on vitamin K antagonist therapy and were therefore excluded. The remaining 410 AF patients on NOAC therapy were approached for participation in the study. However, 228 of these patients (55.6%) had no grandchildren between 5 and 15 years old; 43 patients (10.5%) had grandchildren in the right age category, but did not have a tablet, mobile phone or computer; and another 25 patients (6.1%) were excluded for other reasons.

Of the remaining 114 eligible AF patients, only 15 (13.2%) were willing to participate in the study. Main reasons cited by the 99 patients (mean age 70.0 [SD 6.2] years) for not participating were no interest to participate in general or in the concept in particular (29/99, 29%), not feeling comfortable using technology (22/99, 22%), no interest by the grandchildren or their parents (20/99, 20%), or too busy a lifestyle (12/99, 12%).

The study population of 15 AF patients had a mean age of 69.2 (SD 3.7) years (Table 1). A portable computer (9/21, 43%) and a tablet (9/21, 43%) were mostly used to play with the Health Buddies app. All patients together had 46 eligible grandchildren between 5 and 15 years old, of whom 20 participated in this project (mean age 9.5 [SD 3.0] years old). One patient initiated a contract with 3 grandchildren and 3 patients used the app together with 2 grandchildren. Nine patients were taking a twice daily NOAC (4 on apixaban and 5 on dabigatran). Six patients were taking rivaroxaban, a once daily NOAC. Almost half of the patients (7/15, 47%) used no pill organizer for their medication.

**Table 1:** 

Characteristics		All AF patients (n=15)
Age, mean (SD)		69.2 (3.7)
Male, n (%)		10 (66.7)
**Highest level of education completed, n (%)**		
	Primary school	1 (6.7)
	Secondary school	8 (53.3)
	College or university	6 (40.0)
		
**Kind of AF, n (%)**		
	Paroxysmal AF	4 (26.7)
	Persistent AF	9 (60.0)
	Permanent AF	2 (13.3)
CHA_2_ DS_2_-VASc score^a^, mean (SD)		2.9 (1.5)
HAS-BLED score^b^, mean (SD)		1.3 (0.8)
		
**Time since AF diagnosis, n (%)**		
	<1 year	1 (6.7)
	1-5 years	8 (53.3)
	>5 years	6 (40.0)
		
**Married/cohabiting, n (%)**		
	Yes	15 (100.0)
	No	0 (0.0)
		
**Used electronic device^c^, n (%)**		
	Portable computer	9 (42.9)
	Tablet	9 (42.9)
	Mobile phone	3 (14.2)
		
Total number of eligible grandchildren per patient, mean (SD)		3.1 (1.9)
		
Total number of included grandchildren per patient^d^, mean (SD)		1.3 (0.6)
		
**Age included grandchildren, n (%)**		
	<10 years	9 (45.0)
	≥10 years	11 (55.0)
		
**Dosing regimen NOAC, n (%)**		
	Once daily	6 (40.0)
	Twice daily	9 (60.0)
		
**Time since start NOAC, n (%)**		
	<6 months	2 (13.3)
	6 months-2 years	7 (46.7)
	>2 years	6 (40.0)
		
		
		
**Using a pill organizer, n (%)**				
	Day box	1 (6.7)
	Week box	7 (46.7)
	No	7 (46.7)
Number of medications each day, mean (SD)		5.9 (3.0)	
		
Number of pills each day, mean (SD)		7.0 (3.8)
	

^a^The CHA_2_ DS_2_-VASc score calculates the stroke risk for patients with atrial fibrillation.

^b^The HAS-BLED score estimates the risk of major bleeding for AF patients on anticoagulation therapy.

^c^Some patients used more than one electronic device to use this app (n=21). ^d^Some patients played the game with more than 1 grandchild; 20 grandchildren used the app.

### Motivation to Use the App

Of the 15 patients who started the study and set up the agreement, 13 (87%) completed the contract of 90 days. One patient had technical difficulties using the app, and the other patient was eventually not willing to use the app because the grandchild did not use it.

**Figure 5:**
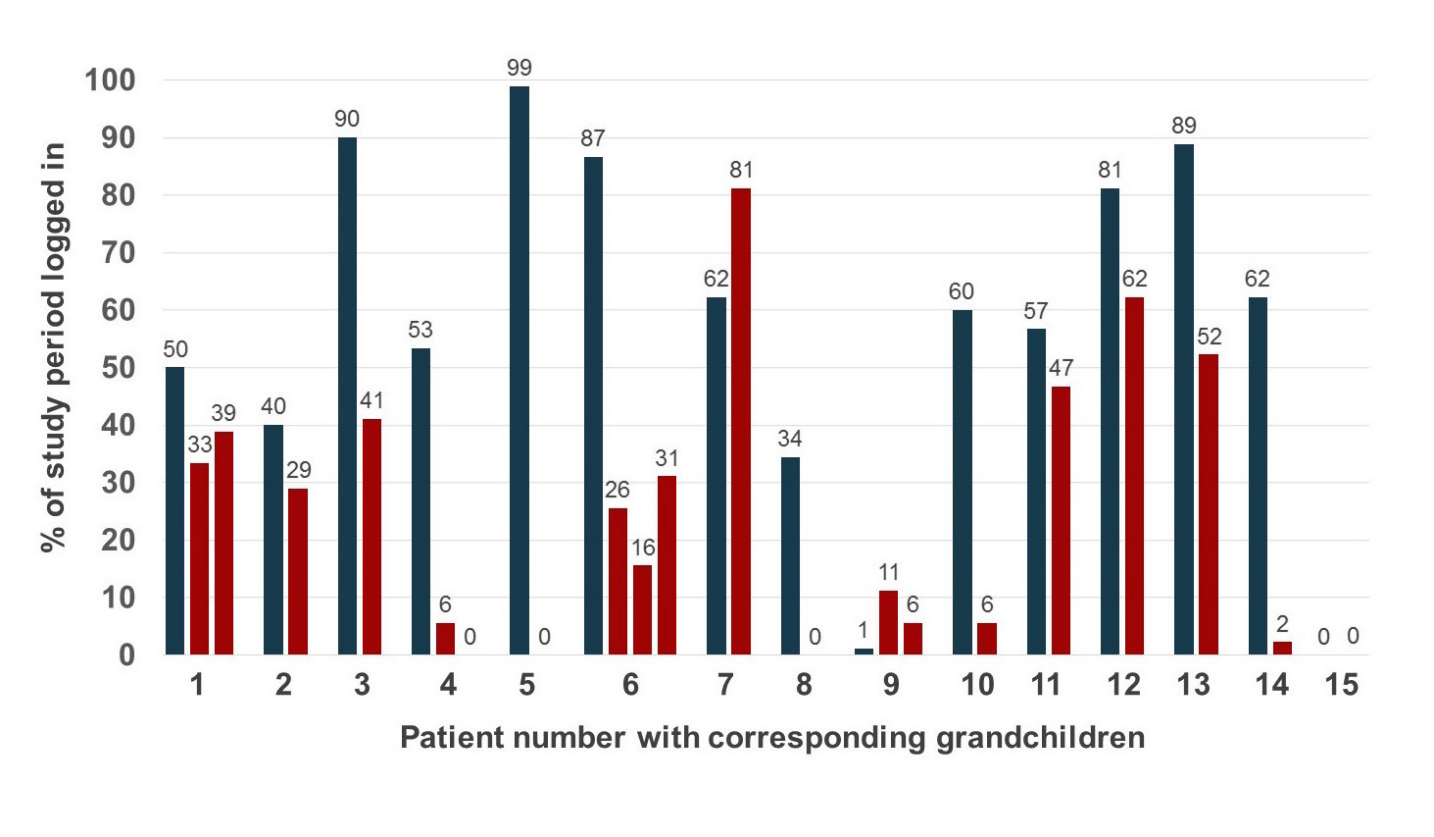
Average percentage of the days logged in to the app by the patients (blue) and grandchildren (red) over the study period of 90 days.

**Figure 6:**
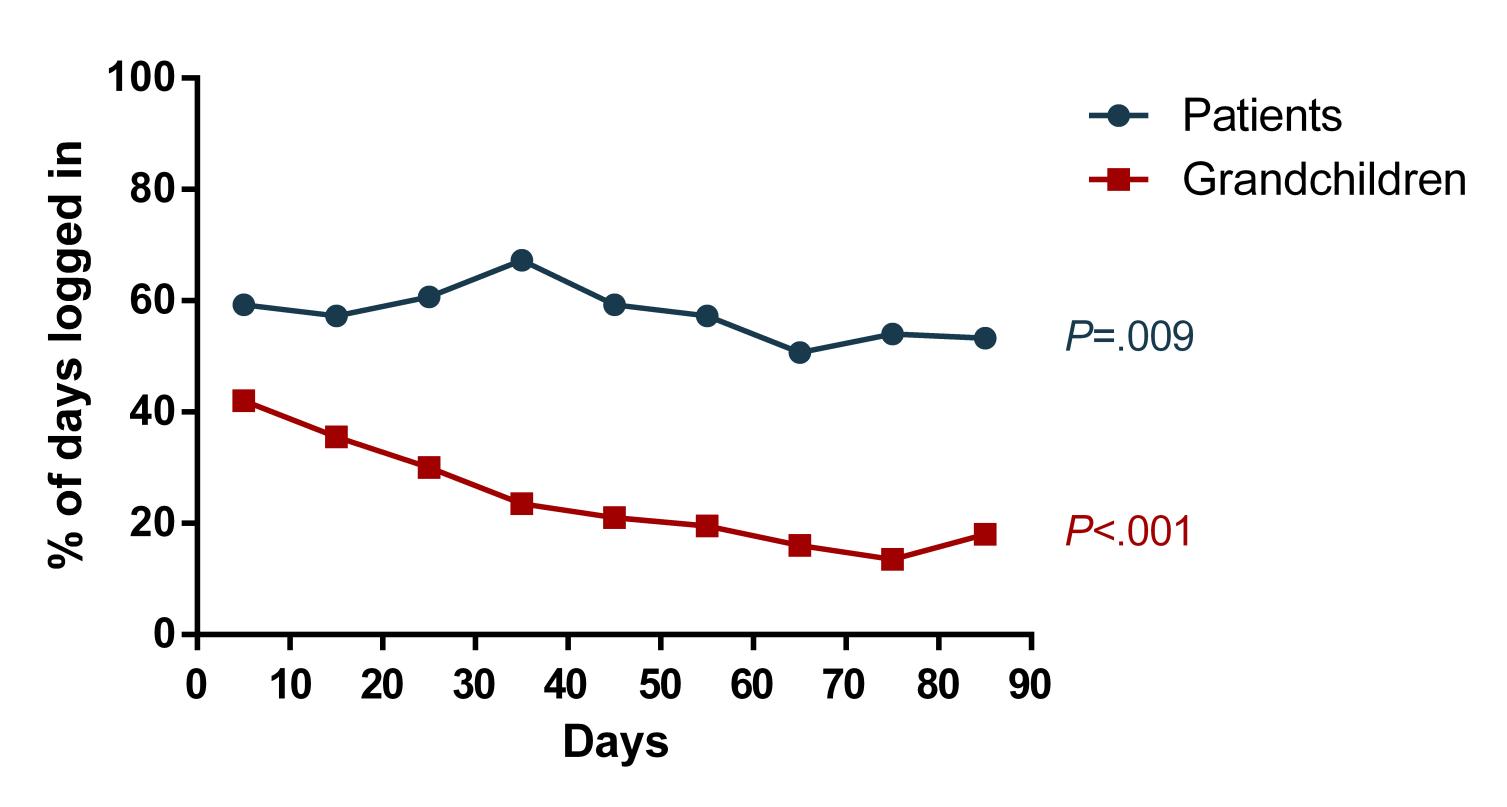
Percentage of the total study period logged in to the app by the patients (blue, n=15) and the corresponding grandchildren (red, n=20).

The frequency of app use after signing the contract differed widely among patients and grandchildren, with the proportion of days logged in to the app ranging from 0%-99% (Figure 5). Mean percentage of days logged in was significantly higher in patients compared to grandchildren (57.7% [SD 30.0] and 24.3% [SD 23.8], respectively; *P*=.002). A weak correlation was found between app use by the patients and their grandchildren (*r*=.37, *P*=.11). Main reasons given not to log in on a daily basis were forgetfulness, holidays, technical problems with the app, hospital admission, not using an electronic device daily, health issues, and the grandchild not using the app. App use significantly decreased towards the end of the study period in both patients (*P*=.009) and grandchildren (*P*<.001) (Figure 6).

### Effects of the App

Patients who completed the contract indicated that they correctly completed their challenge (ie, took their NOAC medication) 99.0% [SD 1.8] of the time. However, electronic monitoring of the medication adherence in 10 patients showed a lower taking adherence and regimen adherence of 88.6% (SD 15.4) and 81.8% (SD 18.7), respectively. Pill count revealed an adherence percentage of 94.5% (SD 9.2). Patients had an average MMAS-8 score of 7.7 (SD 0.6) at baseline and 7.4 (SD 0.9) at the end of the study period (*P*=.44). The percentage of logins to the app over a 3-month period was not significantly correlated with any of the adherence measurements.

At the start of the study, a fifth of patients (3/15, 20%) indicated that they did not know they were diagnosed with AF. After using the app, all patients were aware of their personal medical condition named atrial fibrillation (*P*=.07). The overall score on the JAKQ improved from 64.6% (SD 14.7) at baseline to 70.4% (SD 10.4) after 3 months (*P*=.09).

After signing the contract, 2 patients used the app alone (ie, without their grandchildren), as the grandchild (15 years old) of one patient felt too old to use the app and the grandchild of the other patient was not able to use the app on their device. Of the 13 patients who started to use the app together with their grandchildren, 5 patients (38%) indicated that the use of the app improved their relationship with their grandchildren.

### Patient Experience With the App

Based on the UEQ, patients who played the game and completed the contract (n=13) rated the Health Buddies app positively on clarity (1.500), novelty (0.942), stimulation (0.923), and attractiveness (0.859). Efficiency (0.577) and dependability (0.481) got a neutral evaluation.

Four patients (4/15, 27%) indicated that they would like to use the app together with their grandchild for another period of 3 months. Of these patients whose contract was restarted, only one completed a second 90-day period. Five patients (5/15, 33%) indicated that they would use the app for a second time, but their grandchild would not. The remaining six patients (6/15, 40%) did not want to use the app again. Ten out of 15 patients (67%) found the app easy to use, whereas the remaining 33% (5/15) often encountered technical difficulties or problems. Most patients (11/15, 73%) indicated that the educational aspect of the app was one of its most positive facets. Seven of the 13 patients (54%) using the app together with their grandchildren indicated that their grandchildren liked to play the app.

Although only 1 patient (1/15, 7%) indicated that the app helped to improve his NOAC adherence, 6 patients (6/15, 40%) stated that the project made them more conscious about strict medication adherence and motivated them to be more correct in taking their medication in the future. However, the majority of the patients (8/15, 53%) indicated that they already had very good adherence to their NOAC therapy.

Almost two-thirds of the patients (9/15, 60%) found it useful to receive reminders when they did not play the app, 27% of the patients (4/15) indicated that they never received a reminder, and the minority (2/15, 13%) indicated that they did not like the reminders. Patients suggested broadening the educational aspect as this was a positive feature of the app. They also indicated that their grandchildren would be happy with a larger variety of mini-games and with an adjustment of the difficulty of the mini-games to the age of the grandchild. Some patients suggested adding an alarm function to the app that automatically reminds them to take their medication. However, this is possible only when they use the app on their tablet or mobile phone, which was the case in about half of the patients.

## Discussion

### Principal Considerations

With the increasing number of patients having access to mobile phones, tablets, personal computers, etc, novel methods using these technologies can be used to improve medication adherence and overall management of patients with chronic diseases. In AF patients receiving NOACs, strict medication adherence should be stimulated and ensured to provide an optimal thromboembolic prevention [12,21].

### Usability of the Health Buddies App

The Health Buddies app tries to make therapy adherence fun and stimulating for the patients. However, only 13.7% of the screened AF patients were eligible for inclusion and only 13.2% of those eligible patients were interested in participating. Overall, only 3.7% of the NOAC-taking AF population was included in this project.

More than half of the patients were not eligible as they did not have grandchildren in the right age category. At the end of the study, 60% of the patients were willing to use the app again, but 56% of those indicated that their grandchild would not use the app for a second time. These figures indicate that the target group of patients able to use this app needs to be expanded. However, the concept of a social contract, with completion of challenges between the AF patient and their health buddy, seems valid. With some adjustments (ie, matching the content of the app to the specific health buddy or adding more informative content and reducing the mini-game aspect), it could be possible to involve other health buddies in this app, for example, the patient’s spouse, other family members, friends, or even other AF patients.

Modifications are also necessary to make the app more varied, stimulating, and challenging as app use was lower than expected and decreased over time in patients but especially in grandchildren. The app was developed to target (newly diagnosed) AF patients initiating NOAC therapy to provide them with extra education, to make them conscious about the importance of good adherence, and to create a habit of taking their medication strictly as prescribed. Therefore, the app in its current form was not intended to be used long term, explaining some of the decreased app use over time together with the fact that patients aged >65 years do not typically use their mobile devices and computers as often as younger generations. The majority of the included patients were prescribed NOAC therapy for many months before inclusion, which meant that most of those patients already developed suitable adherence strategies and habits. Furthermore, about half of those patients had a pill organizer to help them adhere. The pilot trial also revealed that it is important to keep the grandchildren motivated to use the app more often, for example, by adjusting the content and difficulty of the app to the age of the grandchildren.

Not all patients seemed to be ready for the innovative concept of this app, mostly because they were not interested and not familiar with the technology. Nevertheless, after playing, patients rated the app positively based on the user experience questionnaire and indicated that the educational aspect was a capital gain.

### The Effect of Health Buddies on Adherence to Non-Vitamin K Antagonist Oral Anticoagulants

Only one patient indicated that the app improved his adherence, although 40% of the patients became more conscious about strict medication adherence. Interestingly, the majority of the patients indicated that they already had very good adherence to their NOAC therapy, also reflected in the self-reported Morisky scale with a mean patient score of 7.7 (out of 8) at the start of the study. It is known that the MMAS-8 often overestimates actual adherence [1]. Electronic monitoring is a more accurate manner to assess medication adherence to NOACs, and it showed a taking adherence of only 88.6% and a regimen adherence of 81.8% in our study. Intriguingly, in the app, patients indicated that they took their NOAC medication 99.0% of the time. Therefore, self-reported adherence through the app is clearly an unreliable way to follow patient adherence. In general, a possible pitfall of the Health Buddies app as well as other adherence promoting apps is that they may only encourage participants to use the app. Equally, they need to motivate them to be adherent to their medication.

Interventions to improve adherence to NOACs are scarce, although it has been shown that nonadherence to NOACs affects health care costs, morbidity, and mortality in the aging AF population [22]. Up until now, there have been only three interventions tested. First, the AEGEAN study investigated the effect of education (ie, booklets and the availability of reminder tools) together with telephone follow-up by a virtual clinic on adherence to apixaban. However, AEGEAN did not find any difference in electronically measured adherence between the usual care group and the intervention group with an adherence value of respectively 88.5% and 88.3% after 24 weeks [23]. Another study by Shore et al, although not prospective, showed that enhanced pharmacist involvement with a longer monitoring and follow-up of patients was associated with an improved adherence to dabigatran [24]. Third, a prior study by our group showed that daily telemonitoring of medication intake with direct personalized telephone feedback led to very high NOAC adherence values with a taking adherence and regimen adherence of 99.0% and 96.8%, respectively [25].

### Educational and Other Effects

The Health Buddies app is also an educational game as patients receive facts and quizzes about AF and the associated therapy. This new way of providing education is needed as different studies showed that the knowledge of AF patients about their arrhythmia and its treatment is low [20,26-31]. Included patients had a mean score on the JAKQ of 64.6% at the start of the study, which is already higher than the score of the average AF patient (ie, 55.8%) [20]. Use of the app led to a small further increase in knowledge level with 5.8%. Moreover, after 3 months, all patients were aware of their heart rhythm disorder, which was not the case for 3 patients at the start of the study.

Increasing patient knowledge seems to be a logical pathway to contribute to better medication adherence and improved overall management [32,33]. However, finding a successful intervention to optimize the knowledge of AF patients is not easy as different interventions were tested with mixed results [20,27,29,30,34]. Most studies used information booklets or educational videos and did not show any significant effect of the intervention [29,30,34]. Only two studies using personalized education found a significant increase in knowledge level [20,27].

Another aim of the app was to strengthen the relationship between patients and their grandchildren. At the end of the study, this was also positively evaluated for about 1 in 3 AF patients.

Finally, the app allowed AF patients to stay in contact with their health care provider by sending emails. We noted, however, that during the 3-month study period, this feature was used only by patients to discuss possible technical difficulties concerning the app.

### mHealth to Improve Adherence

Medication adherence can be addressed in many ways, including automatic reminders, reminder packaging, medication boxes, device aids, counselling, telephone support, patient education, etc, or a combination of those [2,35-37]. It remains unclear which interventions are most effective in improving medication adherence in chronic conditions, and it is especially difficult to prove their effect on clinical outcomes [2,37,38]. Ongoing technological advancements have led to the use of telehealth, eHealth, and mHealth in different domains of health care including medication adherence. Especially mHealth with different mobile apps is being increasingly explored due to its popularity, its portability, and the reachability of a large proportion of the population.

The Health Buddies app was the first mHealth intervention being tested in AF patients to improve adherence to NOACs. In other chronic diseases and also in some cardiovascular illnesses (eg, hypertension and ischemic heart disease), the use of mHealth showed early but promising results in improving medication adherence [3,4,6]. A review by Anglada-Martinez showed that 65% of the mHealth interventions using text messages to send reminders or motivational content found a positive impact on adherence [6]. Another systematic review found that 83% of trials using mHealth technologies in cardiovascular diseases were able to improve adherence and 54% could improve clinical outcomes [4]. The results of most mHealth studies should be interpreted with caution as many interventions used only self-reported adherence to investigate possible improvements in adherence.

However, challenges with mHealth remain as it is not clear which interventions are the most promising, suitable, user-friendly, secure, cost-effective, and how they should best be integrated in daily care [39]. Only by extensive testing of apps and incorporating patients in this process of development and elaboration of the app, as we did with Health Buddies, can these challenges be addressed.

### Study Limitations

An important limitation of this study was the small number of motivated study participants, already having good adherence and acceptable patient knowledge. Moreover, no control group was considered as it was still a pilot study. Nevertheless, the findings from this pilot project provide new insights in the development, usability, and feasibility of the Health Buddies app and mHealth in general for AF patients taking OAC therapy. Other possible limitations are that there were no baseline adherence data gathered with electronic monitoring before patients started using the app and that the study was performed in only one large tertiary care hospital.

### Possibilities for Future Improvements

Even though the Health Buddies app was promising before the start of the pilot study receiving positive reactions during the workshop, it turned out that the usability was low and effects on adherence and knowledge improvement were only limited. Therefore, already suggested adjustments can lead to an upgraded, more accessible, and more effective version of the app. Although patients were already able to include more than one challenge, the app can be made more user-friendly allowing patients to include their entire medication schedule with the possibility of activating appropriate daily reminder alarms. This aspect was not yet incorporated in the app as most patients in the workshops indicated that only occasional and no daily reminders were needed. Other features that can be integrated are the ability of the app to capture overdoses and to allow patients to check for other drug interactions. Another option to broaden the target group is to make a version of the app that can be used individually, although then the Health Buddy concept has to be abandoned and the app should be targeted more on reminders, education, and communication with health care providers. An updated version of the app can be tested in a new pilot study or in a larger prospective randomized controlled trial with the ultimate goal to improve health outcomes in AF patients. Studies could also investigate if the Health Buddies concept can be applied to other chronic diseases.

Still, other new interventions, strategies, and technologies to enhance long-term adherence to NOACs need to be developed and investigated as AF patients are a large and diverse patient population and not all have access to newer mHealth tools. Nonadherence behavior is often multifactorial indicating the necessity of providing patients with tailored, personalized tools.

### Conclusions

The innovative Health Buddies app, based on a social contract concept between AF patients and their grandchildren, was perceived as clear, novel, attractive, stimulating, and educational by its users. However, only a small proportion of the current AF population treated with NOACs seems eligible or is willing to use the app in its current form. Modifications to the app can expand the target group and make it even more motivational and attractive, so that it can be used by more patients and for a longer period of time. That will allow an evaluation of its impact beyond education, that is, on adherence and clinical outcomes.

## Abbreviations

AF: atrial fibrillation
JAKQ: Jessa Atrial fibrillation Knowledge Questionnaire
MEMS: Medication Event Monitoring System
MMAS-8: 8-item Morisky Medication Adherence Scale
NOAC: non-vitamin K antagonist oral anticoagulant
OAC: oral anticoagulation
SD: Standard deviation
UEQ: User Experience Questionnaire

## Appendix

Multimedia Appendix 1 CONSORT-EHEALTH checklist V1.6.1 [15].

## References

[1] Osterberg L, Blaschke T (2005). Adherence to medication. N Engl J Med.

[2] Nieuwlaat R, Wilczynski N, Navarro T, Hobson N, Jeffery R, Keepanasseril A, Agoritsas T, Mistry N, Iorio A, Jack S, Sivaramalingam B, Iserman E, Mustafa RA, Jedraszewski D, Cotoi C, Haynes RB (2014). Interventions for enhancing medication adherence. Cochrane Database Syst Rev.

[3] Gandapur Y, Kianoush S, Kelli HM, Misra S, Urrea B, Blaha MJ, Graham G, Marvel FA, Martin SS (2016). The role of mHealth for improving medication adherence in patients with cardiovascular disease: a systematic review. Eur Heart J Qual Care Clin Outcomes.

[4] Hamine S, Gerth-Guyette E, Faulx D, Green BB, Ginsburg AS (2015). Impact of mHealth chronic disease management on treatment adherence and patient outcomes: a systematic review. J Med Internet Res.

[5] Linn AJ, Vervloet M, van Dijk L, Smit EG, Van Weert JC (2011). Effects of eHealth interventions on medication adherence: a systematic review of the literature. J Med Internet Res.

[6] Anglada-Martinez H, Riu-Viladoms G, Martin-Conde M, Rovira-Illamola M, Sotoca-Momblona JM, Codina-Jane C (2015). Does mHealth increase adherence to medication? Results of a systematic review. Int J Clin Pract.

[7] Haase J, Farris KB, Dorsch MP (2017). Mobile Applications to Improve Medication Adherence. Telemed J E Health.

[8] Obamiro KO, Chalmers L, Bereznicki LR (2016). A Summary of the Literature Evaluating Adherence and Persistence with Oral Anticoagulants in Atrial Fibrillation. Am J Cardiovasc Drugs.

[9] Haim M, Hoshen M, Reges O, Rabi Y, Balicer R, Leibowitz M (2015). Prospective national study of the prevalence, incidence, management and outcome of a large contemporary cohort of patients with incident non-valvular atrial fibrillation. J Am Heart Assoc.

[10] Kirchhof P, Benussi S, Kotecha D, Ahlsson A, Atar D, Casadei B, Castella M, Diener H, Heidbuchel H, Hendriks J, Hindricks G, Manolis AS, Oldgren J, Popescu BA, Schotten U, Van Putte B, Vardas P, Agewall S, Camm J, Baron Esquivias G, Budts W, Carerj S, Casselman F, Coca A, De Caterina R, Deftereos S, Dobrev D, Ferro JM, Filippatos G, Fitzsimons D, Gorenek B, Guenoun M, Hohnloser SH, Kolh P, Lip GY, Manolis A, McMurray J, Ponikowski P, Rosenhek R, Ruschitzka F, Savelieva I, Sharma S, Suwalski P, Tamargo JL, Taylor CJ, Van Gelder IC, Voors AA, Windecker S, Zamorano JL, Zeppenfeld K (2016). 2016 ESC Guidelines for the management of atrial fibrillation developed in collaboration with EACTS. Europace.

[11] Kirchhof P, Ammentorp B, Darius H, De Caterina R, Le Heuzey JY, Schilling RJ, Schmitt J, Zamorano JL (2014). Management of atrial fibrillation in seven European countries after the publication of the 2010 ESC Guidelines on atrial fibrillation: primary results of the PREvention oF thromboemolic events - European Registry in Atrial Fibrillation (PREFER in AF). Europace.

[12] Heidbuchel H, Verhamme P, Alings M, Antz M, Diener HC, Hacke W, Oldgren J, Sinnaeve P, Camm AJ, Kirchhof P (2015). Updated European Heart Rhythm Association Practical Guide on the use of non-vitamin K antagonist anticoagulants in patients with non-valvular atrial fibrillation. Europace.

[13] Blaschke TF, Osterberg L, Vrijens B, Urquhart J (2012). Adherence to medications: insights arising from studies on the unreliable link between prescribed and actual drug dosing histories. Annu Rev Pharmacol Toxicol.

[14] Laliberté F, Nelson WW, Lefebvre P, Schein JR, Rondeau-Leclaire J, Duh MS (2012). Impact of daily dosing frequency on adherence to chronic medications among nonvalvular atrial fibrillation patients. Adv Ther.

[15] Eysenbach G, CONSORT-EHEALTH Group (2011). CONSORT-EHEALTH: improving and standardizing evaluation reports of Web-based and mobile health interventions. J Med Internet Res.

[16] Laugwitz B, Held T, Schrepp M (2008). Construction and Evaluation of a User Experience Questionnaire.

[17] Morisky DE, Ang A, Krousel-Wood M, Ward HJ (2008). Predictive validity of a medication adherence measure in an outpatient setting. J Clin Hypertens (Greenwich).

[18] Krousel-Wood M, Islam T, Webber LS, Re RN, Morisky DE, Muntner P (2009). New medication adherence scale versus pharmacy fill rates in seniors with hypertension. Am J Manag Care.

[19] Morisky Donald E, DiMatteo M Robin (2011). Improving the measurement of self-reported medication nonadherence: response to authors. J Clin Epidemiol.

[20] Desteghe L, Engelhard L, Raymaekers Z, Kluts K, Vijgen J, Dilling-Boer D, Koopman P, Schurmans J, Dendale P, Heidbuchel H (2016). Knowledge gaps in patients with atrial fibrillation revealed by a new validated knowledge questionnaire. Int J Cardiol.

[21] Suryanarayan D, Schulman S (2014). When the rubber meets the road: adherence and persistence with non-vitamin K antagonist oral anticoagulants and old oral anticoagulants in the real world-a problem or a myth?. Semin Thromb Hemost.

[22] Yao X, Abraham NS, Alexander GC, Crown W, Montori VM, Sangaralingham LR, Gersh BJ, Shah ND, Noseworthy PA (2016). Effect of Adherence to Oral Anticoagulants on Risk of Stroke and Major Bleeding Among Patients With Atrial Fibrillation. J Am Heart Assoc.

[23] Montalescot G (2015). Assessment of an education and guidance program for apixaban adherence in non-valvular atrial fibrillation: the randomized AEGEAN study.

[24] Shore S, Ho PM, Lambert-Kerzner A, Glorioso TJ, Carey EP, Cunningham F, Longo L, Jackevicius C, Rose A, Turakhia MP (2015). Site-level variation in and practices associated with dabigatran adherence. JAMA.

[25] Desteghe L, Vijgen J, Dilling-Boer D, Koopman P, Schurmans J, Dendale P, Heidbuchel H (2016). Telemonitoring-based feedback improves adherence to non-vitamin K antagonist oral anticoagulant intake in patients with atrial fibrillation. Eur J Prev Cardiol.

[26] Amara W, Larsen TB, Sciaraffia E, Hernández Madrid A, Chen J, Estner H, Todd D, Bongiorni MG, Potpara TS, Dagres N, Sagnol P, Blomstrom-Lundqvist C (2016). Patients' attitude and knowledge about oral anticoagulation therapy: results of a self-assessment survey in patients with atrial fibrillation conducted by the European Heart Rhythm Association. Europace.

[27] Hendriks JML, Crijns HJ, Tieleman RG, Vrijhoef HJ (2013). The atrial fibrillation knowledge scale: development, validation and results. Int J Cardiol.

[28] Xu W, Sun G, Lin Z, Chen M, Yang B, Chen H, Cao K (2010). Knowledge, attitude, and behavior in patients with atrial fibrillation undergoing radiofrequency catheter ablation. J Interv Card Electrophysiol.

[29] Lane DA, Ponsford J, Shelley A, Sirpal A, Lip GY (2006). Patient knowledge and perceptions of atrial fibrillation and anticoagulant therapy: effects of an educational intervention programme. The West Birmingham Atrial Fibrillation Project. Int J Cardiol.

[30] McCabe PJ, Schad S, Hampton A, Holland DE (2008). Knowledge and self-management behaviors of patients with recently detected atrial fibrillation. Heart Lung.

[31] Koponen L, Rekola L, Ruotsalainen T, Lehto M, Leino-Kilpi H, Voipio-Pulkki LM (2008). Patient knowledge of atrial fibrillation: 3-month follow-up after an emergency room visit. J Adv Nurs.

[32] Heidbuchel H, Berti D, Campos M, Desteghe L, Freixo AP, Nunes AR, Roldán V, Toschi V, Lassila R (2015). Implementation of non-vitamin K antagonist oral anticoagulants in daily practice: the need for comprehensive education for professionals and patients. Thromb J.

[33] Clarkesmith DE, Pattison HM, Lip GY, Lane DA (2013). Educational intervention improves anticoagulation control in atrial fibrillation patients: the TREAT randomised trial. PLoS One.

[34] Clarkesmith DE, Pattison HM, Lane DA (2013). Educational and behavioural interventions for anticoagulant therapy in patients with atrial fibrillation. Cochrane Database Syst Rev.

[35] Mahtani KR, Heneghan CJ, Glasziou PP, Perera R (2011). Reminder packaging for improving adherence to self-administered long-term medications. Cochrane Database Syst Rev.

[36] Brown TM, Siu K, Walker D, Pladevall-Vila M, Sander S, Mordin M (2012). Development of a conceptual model of adherence to oral anticoagulants to reduce risk of stroke in patients with atrial fibrillation. J Manag Care Pharm.

[37] Demonceau J, Ruppar T, Kristanto P, Hughes DA, Fargher E, Kardas P, De Geest S, Dobbels F, Lewek P, Urquhart J, Vrijens B (2013). Identification and assessment of adherence-enhancing interventions in studies assessing medication adherence through electronically compiled drug dosing histories: a systematic literature review and meta-analysis. Drugs.

[38] Kripalani S, Yao X, Haynes RB (2007). Interventions to enhance medication adherence in chronic medical conditions: a systematic review. Arch Intern Med.

[39] Tomlinson M, Rotheram-Borus MJ, Swartz L, Tsai AC (2013). Scaling up mHealth: where is the evidence?. PLoS Med.

