# Corrigendum and Editorial Warning Regarding Use of the MMAS-8 Scale (The Health Buddies App as a Novel Tool to Improve Adherence and Knowledge in Atrial Fibrillation Patients: A Pilot Study)

**DOI:** 10.2196/12202

**Published:** 2018-11-30

**Authors:** Lien Desteghe, Kiki Kluts, Johan Vijgen, Pieter Koopman, Dagmara Dilling-Boer, Joris Schurmans, Paul Dendale, Hein Heidbuchel

**Affiliations:** 1 Faculty of Medicine and Life Sciences Hasselt University Hasselt Belgium; 2 Heart Center Hasselt Jessa Hospital Hasselt Belgium; 3 University of Antwerp and Antwerp University Hospital Antwerp Belgium

## Authors’ Corrigendum

The authors of “The Health Buddies App as a Novel Tool to Improve Adherence and Knowledge in Atrial Fibrillation Patients: A Pilot Study” (JMIR Mhealth Uhealth. 2017;5(7):e98) require corrections in relationship to the use of the Morisky Medication Adherence Scale (MMAS-8).

The following text was omitted from the Acknowledgements and should be added:

Use of the MMAS is protected by US copyright laws. Permission for use is required. A license agreement is available from: Donald E Morisky, ScD, ScM, MSPH, Professor, Department of Community Health Sciences, UCLA School of Public Health, 650 Charles E Young Drive South, Los Angeles, CA 90095-1772.

This statement under the section “Feasibility, Data Collection, and Outcome Measures” requires an advisory warning:

A MMAS-8 score of 0-5 indicates a low adherence, 6-7 is medium adherence, and a score of 8 represents a highly adherent patient.

The new text, which includes the warning, reads as follows:

A MMAS-8 score of 0-5 indicates a low adherence, 6-7 is medium adherence, and a score of 8 represents a highly adherent patient. This MMAS-8 scoring and coding criteria is incorrect, and, if used, would invalidate the MMAS-8 results and potentially put patients at risk of harm.

Two additional references should be cited after reference 17 in the following sentence:

First, the self-reported 8-item Morisky medication adherence scale (MMAS-8) was used to get an idea about the adherence level from the viewpoint of the patient [17].

- Krousel-Wood M, Islam T, Webber LS, Re RN, Morisky DE, Muntner P. New medication adherence scale versus pharmacy fill rates in seniors with hypertension. Am J Manag Care 2009 Jan;15(1):59-66.
- Morisky DE, DiMatteo MR. Improving the measurement of self-reported medication nonadherence: response to authors. J Clin Epidemiol 2011 Mar;64(3):255-7; discussion 258.

These will become references 18 and 19, respectively, and all subsequent references will be renumbered accordingly.

The correction will appear in the online version of the paper on the JMIR website on November 30, 2018, together with the publication of this correction notice. Because this was made after submission to PubMed, PubMed Central, and other full-text repositories, the corrected article also has been resubmitted to those repositories.

## Editorial Notice

Authors and journal were forced to publish this correction due to legal threats by Steven Trubow and Donald Morisky from the company MMAS Research LLC, the copyright holder of the instrument. This is unfortunately not an isolated case, as the developers of this scale are known to comb the literature and ask those who used the scale for research to pay for a retroactive license which may cost thousands or tens of thousands of dollars, and to add references to their work [1].

The Committee on Publication Ethics (COPE) has recently discussed the ethics of this type of behavior by copyright holders of scales ("holding authors to ransom in this way") and recommends to emphasize "the fact that this is not good for the advancement of scientific knowledge or in the public interest" [2]. As open access and open science publisher, JMIR Publications couldn't agree more and we remind our authors of our policies and preference for public and free availability of research tools, including questionnaires [3]. We actively discourage use of MMAS and other instruments which are not available under a Creative Commons Attribution license, and encourage our authors to use or develop/validate new instruments. We are also hereby issuing a special call for papers for short paper instruments or electronic tools licensed under Creative Commons or available under an Open Source license that can be used instead of MMAS to measure medication adherence, and will waive the article submission fee for such development and validation papers describing new instruments that can be used as a free alternative to MMAS.
